# Expanding the Role of Complement Therapies: The Case for Lupus Nephritis

**DOI:** 10.3390/jcm10040626

**Published:** 2021-02-07

**Authors:** Nicholas L. Li, Daniel J. Birmingham, Brad H. Rovin

**Affiliations:** Department of Internal Medicine, Division of Nephrology, The Ohio State University, Columbus, OH 43210, USA; Dan.Birmingham@osumc.edu (D.J.B.); Brad.Rovin@osumc.edu (B.H.R.)

**Keywords:** lupus nephritis, complement, systemic lupus erythematosus, glomerulonephritis

## Abstract

The complement system is an innate immune surveillance network that provides defense against microorganisms and clearance of immune complexes and cellular debris and bridges innate and adaptive immunity. In the context of autoimmune disease, activation and dysregulation of complement can lead to uncontrolled inflammation and organ damage, especially to the kidney. Systemic lupus erythematosus (SLE) is characterized by loss of tolerance, autoantibody production, and immune complex deposition in tissues including the kidney, with inflammatory consequences. Effective clearance of immune complexes and cellular waste by early complement components protects against the development of lupus nephritis, while uncontrolled activation of complement, especially the alternative pathway, promotes kidney damage in SLE. Therefore, complement plays a dual role in the pathogenesis of lupus nephritis. Improved understanding of the contribution of the various complement pathways to the development of kidney disease in SLE has created an opportunity to target the complement system with novel therapies to improve outcomes in lupus nephritis. In this review, we explore the interactions between complement and the kidney in SLE and their implications for the treatment of lupus nephritis.

## 1. Introduction: The Yin and Yang of Complement Protection Versus Injury

Complement is a rapid and effective innate immune surveillance system that acts as a defense against bacterial pathogens, eliminates apoptotic cells and cellular debris, and bridges the innate and adaptive immune systems. Deficiencies in complement proteins predispose to certain infections, for example with encapsulated organisms. Deficiencies in complement components, especially those that result in accumulation of cellular debris, also predispose to autoimmune diseases. Furthermore, to accomplish many of its protective actions, the complement system leverages other components of the immune system and initiates inflammatory responses. To keep inflammation controlled and beneficial, the complement cascade is tightly regulated. Triggers that upset the balance between activation and regulation and/or deficiencies in regulatory mechanisms can lead to uncontrolled inflammation and organ injury. The kidney often bears the consequences of uncontrolled complement activation, especially in the setting of autoimmune diseases. In this review, we will explore the relationship of complement to kidney injury in systemic lupus erythematosus (SLE) and the implications of this relationship for the treatment of lupus nephritis (LN).

## 2. Complement Activation in Three Easy Steps

**Figure 1:**
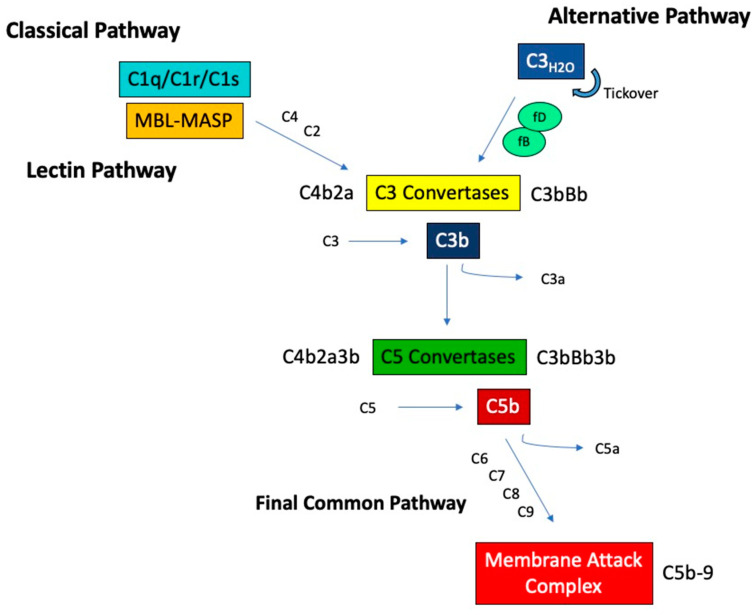
Overview of the complement activation pathways. MBL, mannose-binding lectin, MASP, MBL-associated serine protease.

Complement activation occurs via three distinct pathways that eventually merge into a final common pathway leading to assembly of the membrane attack complex (MAC). These activation pathways, termed the classical, lectin, and alternative pathways, are altogether composed of greater than 30 soluble and membrane bound proteins (Figure 1). Some of these proteins are zymogens, which require cleavage for activation of their enzymatic activity that in turn drives further signaling down the complement cascade.

### 2.1. The Classical Pathway

The classical complement pathway is activated through the binding of C1q to recognition surfaces, such as immune complexes or apoptotic debris. This binding induces a conformational change and the sequential activation of the associated proteases C1r and C1s. Once activated, C1s cleaves C4 into C4a and C4b, creating a binding site on C4b for C2. C2 is then cleaved by C1s, releasing C2b and activating a serine protease site on C2a. This results in a complex, C4b2a, known as the classical C3 convertase, which cleaves C3 into C3a and C3b.

### 2.2. The Lectin Pathway

The lectin pathway is initiated by a member of the collectin family. These molecules are similar to C1q but with C-terminal carbohydrate recognition domains (CRDs) and include mannose-binding lectin (MBL), ficolins, and collectins. Collectins bind mainly to carbohydrates found on pathogens, such as D-mannose and N-acetyl glucosamine, through their CRDs. Once bound, collectins undergo conformational changes that activate MBL-associated serine proteases (MASPs) in a manner analogous to C1r and C1s activation. Activated MASPs can cleave C4 and C2, forming the same C3 convertase as the classical pathway.

### 2.3. The Alternative Pathway

In contrast to the classical and lectin pathways, the alternative pathway is activated via a unique mechanism of spontaneous and continuous hydrolysis of C3, a process known as tickover. Hydrolyzed C3, termed C3_H20_, is able to bind complement factor B (fB), which then is cleaved by the serine protease complement factor D (fD), exposing an active serine protease site on Bb. The resulting complex, C3_H20_Bb, which is stabilized by the complement protein properdin, is the initial alternative pathway C3 convertase, capable of cleaving C3 into C3a and C3b. The formed C3b can bind additional fB, which again can be cleaved by fD, forming the major alternative pathway C3 convertase C3bBb. This feedback activity results in an amplification loop, accelerating C3 activation.

### 2.4. The Final Common Pathway

Regardless of the pathway of initiation, the resulting C3 convertases provide binding sites for additional C3b, forming the complexes C4b2a3b (from the classical or lectin pathways) or C3bBb3b (from the alternative pathway) with specificity for cleavage of C5 into C5a and C5b. When the classical/lectin and alternative pathways are both engaged there is likely more C3bBb3b than C4b2a3b because of the alternative pathway’s amplification loop. C5 cleavage initiates the terminal pathway, where C5b associates with C6 and C7 to form a complex that is capable of inserting into cell membranes via an exposed hydrophobic region on C7. To this membrane bound complex, the addition of C8 and multimers of C9 lead to the formation of the C5b-9 that comprises MAC, which creates pores in the cell membrane causing target cell lysis.

## 3. Complement and LN

### 3.1. Complement Protects against Autoimmunity

The safe clearance of immune complexes (IC), apoptotic cells, and cellular debris is an important protective function of complement [1]. Cellular debris exposes the host to self-antigens and conceivably may foster autoantibody induction. C1q is a potent opsonin for apoptotic cells and its binding to cellular blebs can activate the classical complement pathway resulting in C3 cleavage. Subsequent recognition of C1q bound to cellular debris by its receptor expressed on phagocytes results in engulfment of apoptotic cells. In addition to C1q, MBL is also recognized by this receptor complex as an apoptotic cell opsonin to mediate clearance. The binding of MBL to cellular blebs is through the increased expression of mannose on cells undergoing apoptosis, and this can trigger activation of the lectin pathway. Similarly, ficolins-2 and 3 bind apoptotic bodies and initiate the lectin complement cascade. Uptake and elimination of ficolin-opsonized apoptotic cells is driven by the same C1q receptor interaction on phagocytes. Bound C3 fragments (C3b, iC3b) also contribute to opsonization. C3b and iC3b-opsonized material is recognized by complement receptors 1 through 4 (CR1–CR4) and the complement receptor of the immunoglobulin superfamily (CRIg), which mediate safe processing and removal of this material.

One particular complement-mediated clearance involves the C3b/C4b/C1q receptor CR1 that is expressed on erythrocytes (E-CR1). This form represents >90% of the total circulating CR1. Complement-opsonized ICs bound to E-CR1, a phenomenon known as immune adherence, are prevented from depositing indiscriminately in vulnerable tissue, such as the kidney, brain, and skin, and instead are shuttled to the liver and spleen for safe IC removal [2]. This function appears to protect the kidney during disease flare in LN patients [3].

The importance of complement to protect against the development of SLE and LN is further illustrated by the consequences of genetic defects in complement [4]. Homozygous deficiencies in early components of the classical pathway, including C1q, C1r, C1s, C4, and C2, are reported to increase the likelihood of developing SLE or an SLE-like disease. C1 and C4 deficiency are associated with severe disease manifestations, including LN, in about half of the deficient patients, whereas C2-deficient individuals are more likely to develop skin and joint disease [5,6]. These complement deficiencies are rare, with fewer than 20 reported cases of C1r/C1s deficiency, 30 cases of C4 deficiency and 40 cases of C1q deficiency [7]. The association of SLE and LN with the loss of these classical complement components may be due to impaired IC processing and clearance, reduced removal of apoptotic cells to decrease exposure to self-antigens, or both [8].

As described above, MBL is structurally and functionally similar to classical complement C1q, and genetic alterations leading to deficiency or low serum levels of MBL have also been implicated in SLE [9]. A recent meta-analysis examined the contribution of various polymorphisms in the *MBL-2* gene to SLE susceptibility and found that *MBL-2* variants (alleles *B* and *O*) were associated with increased susceptibility to SLE, while a promoter polymorphism (-221 *X/Y* variant) seemed to provide protection [10]. The characterized coding *MBL-2* polymorphisms are found in Exon 1 and termed alleles *B*, *C*, and *D,* and collectively *O*, and are associated with dramatic reductions in functional serum MBL [11]. The coding region polymorphisms in *MBL-2* are also significantly associated with the development of LN in SLE patients, with the highest risk in those homozygous for combined *O* allele variant, which is associated with complete MBL deficiency [12]. *MBL-2* promoter polymorphisms have been demonstrated to be downregulating, and the *X/Y* variant may confer protection by reducing complement activation and subsequent organ damage [13]. Interestingly, protection was not observed in a meta-analysis with the -550 *H/L* promoter variant of *MBL-2*, which is also downregulating [10].

MBL is an acute phase reactant, which complicates interpretation of its serum levels in inflammatory diseases such as SLE and LN. It has been reported, however, that plasma MBL levels are significantly elevated in SLE patients compared to healthy controls and more pronounced in patients with LN [14]. The susceptibility of patients with deficiencies in MBL implies a protective role for MBL in SLE and LN, similar to what is observed with classical complement. However, the increased expression of MBL in patients with active disease suggests a potential pathologic role as well. In line with this is the observation that a proportion of patients with LN have detectable glomerular MBL deposits [15]. The increase in MBL may be an adaptive response to help mitigate an increase in ICs as SLE and LN begins to flare. At the same time, presentation of increased MBL substrate to the MASPs may fuel damage through the lectin pathway.

Experimental models of complement deficiency support impaired processing of ICs and clearance of cellular debris as causal in the development of autoimmunity. C1q-deficient mice develop high titers of autoantibodies and can develop severe proliferative and crescentic glomerulonephritis, characterized by the presence of glomerular apoptotic bodies, immune deposits, and C3 staining [16,17]. Similarly, C4-deficient mice exhibit spontaneous development of autoantibodies, including anti-dsDNA, along with proliferative glomerulonephritis with mesangial deposition of IgG and C3 [18]. Furthermore, C4-deficient mice demonstrate a delay in the clearance of injected immune complexes from plasma compared to wild-type controls.

### 3.2. Complement Causes Tissue Injury in SLE

Activation of all three complement pathways results in the release of several pro-inflammatory mediators as the pathways proceed toward the generation of MAC. The C3a and C5a fragments of C3 and C5 are anaphylatoxins that promote vasodilation, vascular permeability, and release of reactive oxygen species, histamine, and pro-inflammatory cytokines from leukocytes [19]. C3a and C5a are also chemotactic and recruit macrophages, neutrophils, and B and T cells to sites of tissue inflammation and injury. C3a and C5a act through their receptors C3aR and C5aR1, respectively, to mediate these downstream pro-inflammatory activities. C5a can also engage a second receptor, C5aR2, that may signal opposing events (attenuating C5a effects or promoting inflammation), depending on the circumstances in which it is activated [20].

In health, the complement system is tightly regulated to avoid indiscriminate inflammatory damage to host tissues. The complement cascade has inherent regulation through the natural decay of its convertase enzymes. However, this alone is not sufficient to control complement, so the complement system also relies on a series of proteins that attenuate pathway initiation (C1 inhibitor, C1INH), convertase activity (the regulators of complement activation (RCAs) family), and MAC formation/insertion (vitronectin, CD59, clusterin). The RCAs downregulate C3 activation by either accelerating decay of the convertases, or by serving as cofactors for factor I (fI), a serine protease, which cleaves C4b and C3b and effectively halts convertase activity. Decay acceleration is mediated by C4b binding protein (C4BP), factor H (fH), decay accelerating factor (DAF), and complement receptor type 1 (CR1). The cofactor activity of fI is also mediated by the activities of C4BP, fH, CR1, and membrane cofactor protein (MCP).

The consequences of impaired complement regulation for LN have been demonstrated in murine models of lupus. Knocking out fH in lupus-prone mice resulted in unrestricted alternative pathway activation, hypocomplementemia, accelerated kidney failure with marked albuminuria, and early mortality compared to lupus-prone mice with intact fH [21]. Furthermore, fH-deficient mice rapidly developed severe LN-like glomerulonephritis by 12 weeks of life, with kidney biopsies demonstrating mesangial, endocapillary, and extracapillary proliferation and glomerular IC deposits, akin to LN in humans. Conversely, lupus-prone mice that lack proteins needed for alternative pathway activation are protected from the development of renal disease. For example, lupus-prone mice deficient in complement fB or fD have delayed progression of glomerulonephritis with reduced glomerular hypercellularity compared to mice with intact fB and fD [22,23].

The role of complement fH in human LN has been also been investigated. Mutations and polymorphisms in fH are most commonly associated with atypical hemolytic uremic syndrome (aHUS) and age-related macular degeneration (AMD), however links to SLE and glomerulonephritis are also reported [24,25]. Serum levels of fH have also been observed to be significantly reduced in patients with LN compared to individuals with non-renal SLE and healthy controls [26]. Furthermore, when compared between patients with active LN versus LN in remission, those with quiescent disease had significantly higher levels of fH as well. While fH levels were able to differentiate patients with active LN, in this study, there was no significant difference in renal survival at the end of follow up (median 54 months). Finally, in a longitudinal study of the relationship between C3 levels and LN flare, patients homozygous for the same fH polymorphism associated with AMD had an eight-fold risk of flare during C3 consumption, which is more than twice the risk as in patients who are homozygous wildtype for this polymorphism [27]. One interpretation of these data is that during active LN fH is consumed in an attempt to control C3 cleavage and its consequences. The degree of successful control may dictate whether LN develops, and if it develops, how clinically severe it becomes. Patients with fH polymorphisms that render it less effective in controlling C3 cleavage are at increased risk for clinically apparent LN.

These data suggest that uncontrolled alternative pathway activity can lead to or worsen LN. Importantly, several murine studies have shown that specific inhibition of the alternative pathway, even in the presence of intact complement regulatory systems, can attenuate kidney injury in lupus. In lupus-prone mice, targeted reduction in circulating complement fB using antisense oligonucleotides prevented the development of hypocomplementemia, as well as LN-like pathology with reductions in proteinuria and glomerular C3 deposition and improved survival [28]. Similarly, treatment of lupus-prone mice with a soluble CRIg-Fc increased serum C3 levels and significantly improved proteinuria and renal pathology, including a decrease in glomerular IC staining [29]. Selective targeting of the alternative pathway in lupus-prone mice with a recombinant CR2-fH chimera, which targets fH to tissue sites of C3 activation through CR2 binding, likewise reduced the production of anti-dsDNA and circulating ICs, increased serum C3 levels, and prevented the development of albuminuria and glomerular deposition of IgG, C1q, and C3 [30]. Finally, treatment of lupus-prone mice with a soluble C3 inhibitor prevented the development of renal failure, albuminuria, and significantly reduced glomerulosclerosis [31].

In human LN, the contribution of the alternative complement pathway to the development of kidney damage is supported by the observation that reduction in C3, but not C4, is associated with LN flare, and with the development of circulating anti-C3b antibodies [27,32]. Therefore, alternative complement activation at the time of LN flare likely results in C3 activation in kidney tissues causing inflammatory damage and exposes C3b epitopes, which drive the production of anti-complement antibodies.

The terminal complement pathway leading to generation of MAC has also been evaluated in murine LN models. Early studies aimed at preventing the formation of MAC with anti-C5 monoclonal antibody (mAb) in lupus-prone mice demonstrated delayed development of proteinuria and glomerulonephritis and prolonged survival compared to isotype matched mAb [33]. Alternatively, this attenuation of kidney injury may be due to impaired generation of C5a and loss of its pro-inflammatory activity. In humans, MAC staining is strongly associated with LN and is detectable in glomeruli and tubular basement membranes of patients with active as well as chronic disease [34,35].

## 4. Complement as a Therapeutic Target in LN

A plethora of novel therapeutics have been, or are being, developed that inhibit complement activation (Table 1). Given the involvement of complement in the pathogenesis of LN, it is reasonable to consider such therapies for treatment. Targeting complement in LN is not, however, straightforward because of complement’s paradoxical roles in both providing protection by IC processing/clearance and mediating tissue damage. In particular, with the protective effects being associated with the classical and lectin pathways, therapies directed at these complement components would likely be less efficacious and may even be harmful in the treatment of LN. On the other hand, the alternative and common terminal pathways, which are involved in many of the inflammatory activities of the complement system, appear to be potentially high yield therapeutic targets in patients with LN. Selectively inhibiting components or activating regulators of these pathways in LN may prevent the damaging kidney effects of complement without sacrificing the protective components of the classical and lectin cascades.

LN may recur post-kidney transplant, but the incidence of clinically severe LN is low [36,37,38]. Recurrent LN may not significantly decrease patient or allograft survival [36,37]. Several patients with recurrent disease did show hypocomplementemia and glomerular C3 plus C1q in their allograft biopsies, suggesting the pathogenesis of recurrent LN may still involve the complement system [37]. Therapy of patients with recurrent LN should probably be considered on an individual basis. It may be reasonable to target complement in such patients if the LN flare is severe and is threatening the allograft, especially if the LN is recurring despite an adequate anti-rejection regimen using immunosuppressives that would also be used in SLE, but this needs to be tested.

Due to the severity of LN and the fact that it can rapidly damage the kidney, all novel therapies have been added to background, standard of care immunosuppression in clinical trials. At present, the most often used background medications for LN are glucocorticoids, mycophenolate mofetil (MMF) or its derivatives, cyclophosphamide, calcineurin inhibitors (CNIs), and rituximab. The addition of any novel, targeted therapy to background medications should consider how a novel drug will affect the efficacy of standard therapy, and whether a new drug is additive or, by inhibiting the same pathways as standard drugs, redundant. Redundancy may not improve efficacy but may allow reduced dosing of particularly toxic medications. Of course, the risk/benefit ratio of increasing immunosuppression must always be determined.

### Compatibility of Complement Therapy with LN Standard-of-Care

Almost every patient with LN will be treated with a glucocorticoid. In a microarray analysis of peripheral blood mononuclear cells from healthy donors treated with dexamethasone, the transcripts for C1q, C3, and C5 were upregulated, while several complement regulatory transcripts were downregulated [39]. In contrast, glucocorticoid treatment was found to increase monocyte C3 and fB mRNA early, but after 72 h, secretion of C3 and fB fell while fH increased compared to controls [40]. Although these results are somewhat conflicting, they suggest that adding a complement inhibitor to a glucocorticoid regimen could counteract steroid-induced complement expression and possibly synergize with complement inhibition, which may allow a reduction in steroid dosing.

MMF is the most common immunosuppressive agent added to glucocorticoids. In a rodent model of ischemia/reperfusion injury, complement component C3 is upregulated in the kidney. C3 expression was reduced by pre-treatment of the animals with MMF, but to a greater extent, with mycophenolic acid-glucosamine conjugate, which is targeted to the proximal tubule [41]. Hence, by adding a complement inhibitor, there may be potential to enhance the benefits of MMF, which may not target the kidney as well as its acid-glucosamine conjugate. This finding also raises the potential importance of local versus systemic effects of all therapies of LN, alluded to above with the CR2-fH chimera and which is discussed further below.

The effects of cyclophosphamide on complement have been evaluated on guinea pig lung macrophages. After intraperitoneal injections of saline or cyclophosphamide for up to 7 days, it was found that cyclophosphamide inhibited lung macrophage production of C2 and C4 by 63% and 79%, respectively, as early as 24 h after treatment but had no effect on systemic levels [42]. It is not clear whether local tissue inhibition of these early complement components would worsen disease through loss of IC opsonization, but the addition of a late pathway complement inhibitor may synergize with cyclophosphamide in blocking the inflammatory potential of the alternative and terminal pathways.

The use of CNIs have been associated with increases in the expression of complement components. Mice treated for two weeks with cyclosporin A (CsA) developed complement-mediated renal injury as evidenced by glomerular C3 deposition in wild-type mice, which was not observed in fB-deficient mice [43]. Additionally, when renal proximal tubule cell lines were incubated with CsA or tacrolimus, expression of various complement activation components from the classical and alternative pathways were upregulated, while the expression of complement inhibitors was reduced [44]. Therefore, these data suggest that CNIs may enhance the inflammatory effects of complement within the kidney, potentially exacerbating kidney damage in LN. The addition of complement-targeted therapies to CNIs may, thus, be synergistic for the treatment of LN.

Rituximab is an anti-CD20 mAb that depletes B cells via various mechanisms, including complement-dependent cytotoxicity (CDC), antibody dependent cellular cytotoxicity (ADCC), and direct cell death (DCD). Therefore, in diseases where complement dysregulation is a hallmark such as SLE, the efficiency of rituximab may be affected. In a B cell lysis assay, the effectiveness of rituximab was significantly reduced in the presence of complement-depleted serum compared to normal human serum, highlighting the importance of CDC to its mechanism of action [45]. Given its dependence on functional complement, the addition of a complement inhibitor to rituximab therapy may reduce its efficacy.

## 5. Timing Is Everything: When Should a Complement-Targeted Intervention Be Started?

The use of complement-directed therapy is more likely to be successful when given to patients when the inflammatory effects of complement are being activated. While this statement seems self-evident, it is harder to operationalize than would be expected. Complement may be activated systemically and/or locally in the kidney. While systemic complement activation is reflected by peripheral blood hypocomplementemia, it is not clear how well peripheral blood complement measurements reflect what is happening at the tissue level. Although it is also not clear whether systemic or local activity is most important for kidney injury in LN, therapeutics that address both may be relevant. Complement is consumed peripherally in many lupus patients during active disease, and even more so in active LN, but many patients with active, biopsy-proven LN have normal circulating C3 and C4 levels [46]. Patients with active disease also come to clinical attention at different times during their course of LN, and complement activation, especially within the kidney, may vary over time. It would be ideal to have a non-invasive biomarker for intra-renal alternative/terminal pathway activation to guide the timing and use of directed therapy. While such biomarkers are not yet available, possibilities are emerging.

### 5.1. Systemic Complement Activation Markers

The C3 and C4 hypocomplementemia in LN is typically more pronounced in patients with active LN compared to inactive nephritis [47]. One of the problems in assessing C3 and C4 levels is that hypocomplementemia is defined based on the very wide normal ranges in most clinical laboratories. This is particularly relevant for C4, which has extensive gene copy number variation that is reflected in circulating C4 levels [48]. It is conceivable that tracking an individual’s C3 and C4 levels more closely would reveal decreases in the normal range that would normally go unnoticed but do represent complement activation. In lieu of this level of scrutiny, autoantibodies to complement proteins may reflect complement activation and LN activity. Anti-C1q antibodies are found in LN patients and can differentiate active versus quiescent nephritis, albeit with only moderate sensitivity and specificity [49,50]. Anti-C3b antibodies differentiate patients with LN and non-renal SLE with greater specificity and identify LN patients who may be prone to flare [32]. Importantly, antibody titers of anti-C1q and anti-C3b also correlated inversely with circulating C4 and C3, suggesting their relationship to classical and alternative complement activation, respectively.

### 5.2. Intra-Renal Complement Activation Markers

It has been known for decades that complement genes can be expressed locally in the kidney, including C4, C3, and fB [51,52]. More recently, transcriptomic analysis of kidney biopsies from LN patients at renal flare showed intra-renal upregulation of many genes associated with the alternative complement pathway, including C3 and fD, expressed at significantly higher levels than in normal kidney [53]. Following a standard 6-month induction treatment, LN patients that had not achieved a complete or partial clinical renal response exhibited even greater differential expression of C3 and fD than in their flare biopsies. In these repeat biopsies, the fI transcript level fell relative to control, suggesting suppression of this important C3b regulator. In contrast, patients who had achieved a complete clinical renal response showed a decrease in complement transcript expression from the elevated levels of their flare biopsies. Dysregulated gene expression of the alternative complement pathway at LN flare suggests this may be an appropriate time to intervene with a complement-targeted therapy. The persistent abnormal expression of complement transcripts in patients non-responsive to conventional therapy also suggests that complement-targeted treatment may rescue a non-response.

These transcriptomic results reinforce the studies discussed previously that showed a key role of the alternative pathway in LN. This conclusion is also supported by the presence of fB, fH, and properdin in the glomeruli of LN patients, and the deleterious findings associated with their presence. Glomerular staining of fB and fH was associated with more severe interstitial fibrosis, and patients with properdin-positive biopsies had significantly higher proteinuria than those without evidence of intra-glomerular alternative pathway activation [54]. Moreover, patients with biopsies demonstrating glomerular deposition of C3, without C4 or C1q, had a poorer response to therapy at 1 year and were more likely to experience progression of chronic kidney disease [55].

Besides the alternative pathway, LN kidneys have evidence of lectin pathway activation, as evidenced by the detection of glomerular MBL and L-ficolin. Patients positive for lectin pathway proteins are reported to have increased proteinuria compared to those without, although fB and fH were often also detected in these samples [54].

While these biopsy findings may be useful surrogates of intra-renal complement activity, using kidney biopsy complement staining or transcript expression as a biomarker is problematic, as it cannot be assessed regularly or frequently. However, complement activation products have been detected in the urine of patients with a variety of glomerular diseases and may be surrogate markers of intra-renal complement activity, although it can be difficult to exclude filtration of serum complement as a source of urine complement. In SLE, urinary C3 and its fragments are present in patients with active LN [56]. Serum C3d is elevated in patients with SLE compared to healthy controls, while urinary C3d is present only in patients with active SLE, and individuals with LN have significantly higher levels in urine compared to those with non-renal SLE [57]. In addition to C3d, MAC has also been reported to be detectable in the urine of patients with various glomerular diseases, including LN, and may, therefore, be representative of renal terminal complement pathway activation [58].

## 6. Complement Therapeutics Today and Tomorrow

At present, there are few clinical data on the efficacy of complement inhibitors in SLE, LN, or autoimmunity. Eculizumab is a humanized mAb to complement C5 (NCT01221181). It prevents the activation of C5, the subsequent release of C5a and formation of MAC. It is currently approved for the treatment of paroxysmal nocturnal hemoglobinuria (PNH) and aHUS. A systematic review evaluating the role of eculizumab in LN related thrombotic microangiopathy (TMA) was recently published and assessed 14 studies involving 30 patients [59]. The majority of patients in these reports had been initially treated with glucocorticoids and plasma exchange, in addition to various immunosuppressives, including cyclophosphamide, MMF, and rituximab. With eculizumab therapy, 28/30 patients experienced a positive response, defined as renal recovery, resolution of symptoms, or successful hospital discharge. Furthermore, 13 patients were able to discontinue eculizumab, while five remained on therapy at the last follow up.

Eculizumab has also been given to a patient with severe, refractory class IV LN that had not responded to multiple rounds of induction therapy with cyclophosphamide and rituximab and maintenance MMF with and without tacrolimus [60]. Resistant disease was reflected by persistent kidney dysfunction and worsening proteinuria and confirmed by repeat kidney biopsies. Kidney histology confirmed the presence of complement C9, a component of MAC. Eculizumab combined with methylprednisolone was given, followed by MMF maintenance. After receiving eculizumab the patient had a rapid and sustained improvement in kidney function and repeat kidney biopsy 18 months later demonstrated resolution of endocapillary hypercellularity and undetectable C9 staining. Ravulizumab is a novel humanized mAb that, like eculizumab, targets C5 and is also approved for PNH and aHUS. Ravulizumab is currently under investigation in a phase 2 trial of proliferative LN (NCT04564399). Finally, a small interfering RNA targeting C5, ALN-CC5 (cemdisiran) has also been developed and has been investigated in a phase 2 study of PNH (NCT02352493).

Avacopan, a small molecule C5a receptor antagonist, has not been trialed in LN to date but has been tested in anti-neutrophil cytoplasmic antibody (ANCA) vasculitis, another autoimmune disease in which kidney injury appears to be mediated, in part, by complement (NCT02994927). A phase 2 trial demonstrated that avacopan was effective in accelerating a pre-determined BVAS response when added to (mainly) cyclophosphamide [61]. Importantly, this was done in the context of a lower than usual dose of glucocorticoid in one study arm, and no glucocorticoid in the second arm of the trial. The effect of a C5a receptor antagonist is most likely to attenuate inflammation, given the pro-inflammatory effects of C5a. This may have similar efficacy in LN, especially the proliferative forms characterized by significant intra-renal inflammation. 

Narsoplimab is a human mAb against mannan-binding lectin associated serine protease-2 (MASP-2), a critical enzyme involved in the activation of the lectin pathway. A phase 2 trial of narsoplimab to evaluate its effects on proteinuria in LN is underway (NCT02682407).

Although not currently being studied in LN, complement fB and fD inhibitors are being evaluated in other glomerular diseases such as IgA nephropathy and C3 glomerulopathy. These include a small molecule fB inhibitor (NCT03373461), an anti-sense oligonucleotide against factor B (NCT04014335), and a small molecule fD inhibitor (NCT03369236) [62,63]. Targeting fB and fD in LN would also be expected to be effective. APL-2 is a synthetic cyclic peptide inhibitor of C3 and is currently being investigated in a group of glomerular diseases, including LN (NCT03453619).

## 7. Conclusions

The complement system is active in human lupus and LN. Indirect evidence of systemic complement consumption along with complement split product deposition in the kidneys during active LN imply a pathogenic role for complement in LN. In contrast, naturally occurring genetic deficiencies in classical complement components, specifically those that help safely dispose of cellular debris and immune complexes, may lead to the development of LN, suggesting a protective role for complement. Animal models of LN have helped sort out protection from injury by demonstrating that inhibition of pro-inflammatory mediators released during the later stages of complement activation attenuates kidney injury. These findings provide a clear strategy for modulation of the complement system as a therapeutic approach for the treatment of LN. The optimal therapeutic will spare the early components of the classical and lectin pathways, in favor of inhibiting the alternative and terminal pathways, thereby blocking the effects of the pro-inflammatory complement split products, preventing activation of the MAC, or enhancing the intrinsic complement regulatory pathways to the level needed to meet the increases that occur in complement activation. A variety of novel small molecules and biologics have been developed that meet these criteria, and clinical trials are, or shortly will be, underway to test their efficacy. Although the pre-clinical and clinical data reviewed here suggest that a complement-targeted approach should be successful in LN, it must be assessed in the context of adding more immunosuppression to patients whose treatments already render them significantly immunosuppressed.

## Figures and Tables

**Table 1:** Complement-targeted therapies in clinical trials of kidney disease.

Drug Name	Drug Type	Complement Target	Stage	Disease	Clinical Trials Identifier
ACH-4471	Small Molecule Inhibitor	Factor D	Phase 2	C3G	NCT03369236
ALN-CC5	Small Interfering RNA	C5	Phase 2	PNH	NCT02352493
APL-2	Synthetic Cyclic Peptide	C3	Phase 2	Various Glomerulopathies	NCT03453619
Avocopan	Small Molecule Inhibitor	C5aR1	Phase 3	AAV	NCT02994927
Eculizumab	Monoclonal Antibody	C5	Phase 1	C3G	NCT01221181
IFX1	Monoclonal Antibody	C5a	Phase 2	AAV	NCT03712345
IONIS-FB-LRx	Ligand Conjugated Antisense Inhibitor	Factor B	Phase 2	IgAN	NCT04014335
LNP023	Small Molecule Inhibitor	Factor B	Phase 2	IgAN	NCT03373461
Narsoplimab	Monoclonal Antibody	MASP-2	Phase 3 Phase 2	IgAN LN	NCT03608033 NCT02682407
Ravulizumab	Monoclonal Antibody	C5	Phase 2	LN	NCT04564399

C3G, C3 glomerulopathy; PNH, paroxysmal nocturnal hemoglobinuria; AAV, ANCA-associated vasculitis; IgAN, IgA nephropathy; LN, lupus nephritis. Source: ClinicalTrials.gov, (accessed on 31 December 2020).

## Data Availability

Data sharing not applicable.
